# Targeting of interleukin (IL)-17A inhibits PDL1 expression in tumor cells and induces anticancer immunity in an estrogen receptor-negative murine model of breast cancer

**DOI:** 10.18632/oncotarget.13819

**Published:** 2016-12-07

**Authors:** Yun-Feng Ma, Chen Chen, Dongqing Li, Min Liu, Zhuang-Wei Lv, Yanhong Ji, Jiru Xu

**Affiliations:** ^1^ Department of Pathogenic Microbiology and Immunology, School of Basic Medical Sciences, Xi‘an Jiaotong University Health Science Center, Xi’an, P. R. China; ^2^ Clinical School of Hubei University of Chinese Medicine, Hubei University of Chinese medicine, Wuhan, Hubei, China; ^3^ Department of Microbiology, School of Basic Medical Science, Wuhan University, Wuhan, P. R. China; ^4^ Department of Immunology, School of Basic Medical Science, Wuhan University, Wuhan, P. R. China; ^5^ Key Laboratory of Environment and Genes Related to Diseases (Xi’an Jiaotong University), Ministry of Education of China, P. R. China

**Keywords:** interleukin-17, programmed death ligand 1, breast cancer, estrogen receptor, immunotherapy

## Abstract

The expression of IL-17A and programmed death ligand 1 (PDL1) is increased in estrogen receptor-negative breast cancer. IL-17A promotes tumor cell survival and invasiveness and inhibits the antitumor immune response. The PDL1–PD1 (programmed death protein 1) signaling pathway promotes escape from immune surveillance in tumor cells. The pro-tumor properties of IL-17A and PDL1 in various cancers have been previously examined; however, the relationship and roles of IL-17A and PDL1 in ER-negative breast cancer have not been evaluated. Therefore, we assessed whether IL-17A promotes PDL1 expression in tumor cells and whether targeting of IL-17A could inhibit ER-negative breast cancer progression in a murine model. Our study revealed that IL-17A promoted PDL1 expression in human and mouse cells. In the murine cancer model, targeting of IL-17A inhibited PDL1 expression in the tumor microenvironment, decreased the percentage of Treg cells in tumor-infiltrating lymphocytes, and promoted CD4^+^ and CD8^+^ T cells to secrete interferon gamma. More importantly, treatment with combined anti-IL-17A and anti-PDL1 antibodies enhanced antitumor effects in favor of tumor eradication. Thus, our study established a pro-tumor role of IL-17A in promoting tumor immune escape and supports the development of a novel cytokine immunotherapy against breast cancer.

## INTRODUCTION

Breast cancer remains one of the most commonly diagnosed cancers in women worldwide and is the second leading cause of mortality, after lung cancer [1, 2]. Among the various prognostic factors, lack of estrogen receptor (ER) has been consistently associated with poor prognosis [3, 4]. ER-negative breast tumors exhibit high cytokine content [4]; especially, the level of IL-17A is significantly increased [5, 6]. IL-17A is a pro-inflammatory cytokine associated with poor prognosis in breast cancer [5, 7]. Because of the high expression of IL-17 receptor chains on tumor cells, IL-17A has direct effects on these cells [7]. IL-17A promotes tumor cell survival and invasiveness and inhibits the antitumor immune response by interacting with myeloid-derived suppressor cells (MDSCs) [7–9]. Inhibition of IL-17A augments the cytotoxicity of tumor-infiltrating lymphocytes and contributes to tumor suppression in colon cancer and lung cancer in mice [10, 11]. However, the role of IL-17A in ER-negative breast cancer has not been exhaustively evaluated.

The programmed death ligand 1–programmed death protein 1 (PDL1–PD1) signaling pathway induces anergy in tumor-specific T cells by expressing PDL1 on their surface [12, 13]. Inflammatory signals such as IFN-γ in tumor tissues induce the expression of PDL1 [12]. Additionally, the MEK–ERK and PI3K–Akt signaling pathways are involved in PDL1 regulation [14, 15]. It has been reported that IL-17A enhances the phosphorylation of MEK–ERK in breast tumor cells [6]. However, whether IL-17A promotes PDL1 expression remained unclear. In addition, both PDL1 and IL-17A are associated with poor prognosis in breast cancer [5, 16, 17]; thus, targeting of PDL1 and IL-17A might be an effective treatment for breast cancer. Based on these previous findings, we hypothesized that IL-17A may regulate the immune checkpoint molecule PDL1 and that inhibition of IL-17A may elicit an anti-tumor immune response in murine models of ER-negative breast cancer.

In the current study, we explored the mechanisms of PDL1 regulation by IL-17A, and we determined whether targeting of IL-17A and PDL1 could inhibit ER-negative breast cancer progression in mice.

## RESULTS

### IL-17A and PDL1 expression are correlated in ER-negative breast cancer

**Figure 1:**
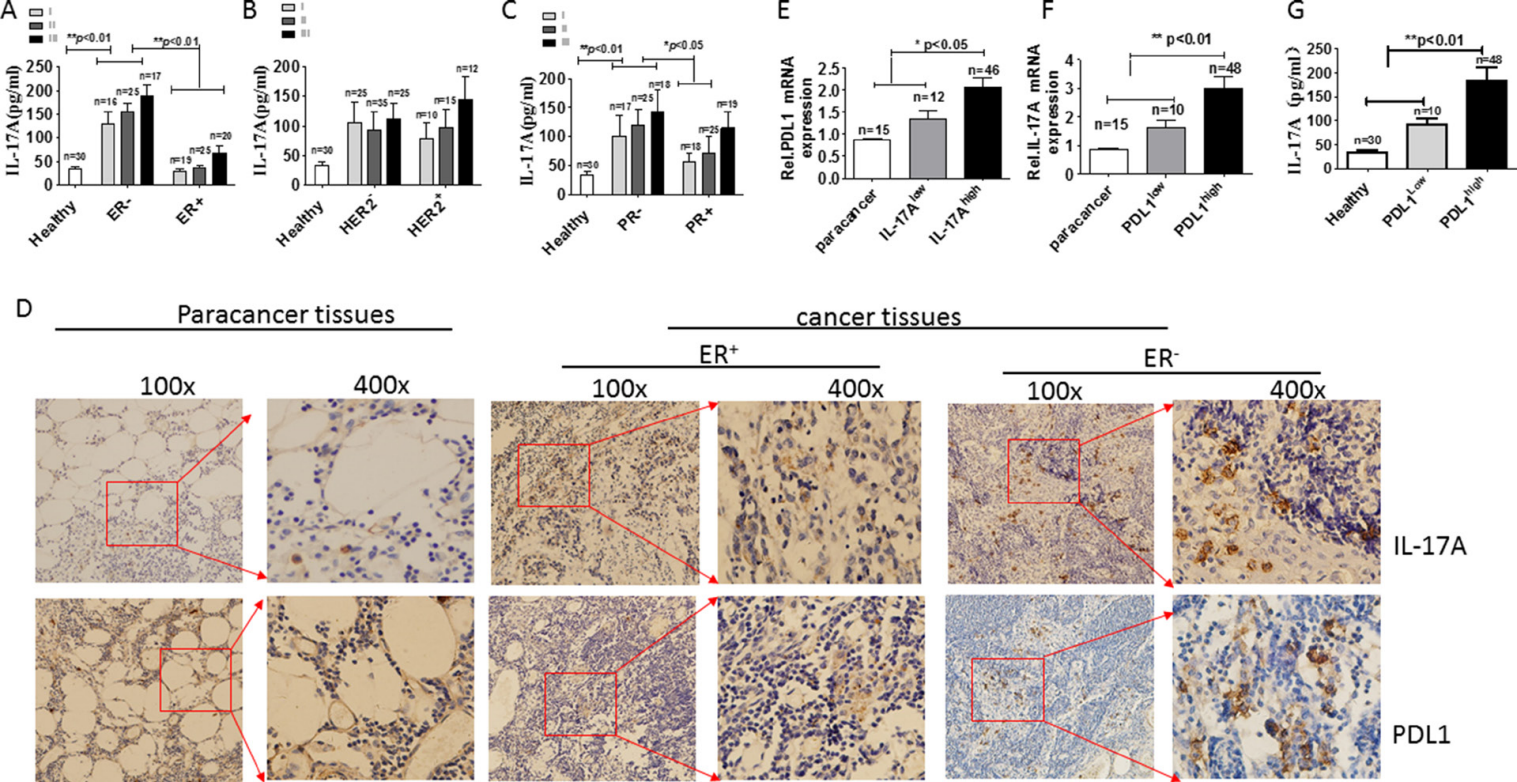
Expression of IL-17A and PDL1 in breast cancer patients Serum IL-17A levels in breast cancer patients with different ER status (**A**), HER2 status (**B**), and PR status (**C**). (**D**) Representative photomicrographs (100× and 400× magnification) of immunohistochemical staining of IL-17A-positive and PDL1-positive tumor tissues from ER-positive or -negative breast cancer patients. (**E**) Quantitative analysis of human *PDL1* mRNA expression in IL-17A^high^ and IL-17A^low^ tumor tissues from ER-negative breast cancer patients. (**F**) Quantitative analysis of human *IL-17A* mRNA expression in PDL1^high^ and PDL1^low^ tumor tissues from ER-negative breast cancer patients. (**G**) ELISA analysis of human serum IL-17 levels in PDL1^high^ and PDL1^low^tumor tissues from ER-negative breast cancer patients. Data are representative of three experiments. Error bars represent SEM.

Cochaud *et al*. [6] reported that IL-17A-producing cells are elevated in breast tumors, and increased IL-17A seemed to be mainly associated with ER-negative tumors. We checked serum IL-17A levels in 122 breast cancer patients and found that IL-17A levels correlated with ER/PR-negative status, but not with HER2 expression and clinical tumor stage (*p* < 0.05, Figure 1A–1C). Next, we assessed the expression of IL-17A and PDL1 in tumor tissue samples by IHC. Strong expression of IL-17A and PDL1 was observed in ER-negative tumors; 79% of ER-negative tumors was infiltrated by IL-17A^high^ cells and 82% by PDL-1^high^ cells (Table 1 and Figure 1D). Conversely, only about 20% of ER-positive tumors contained IL-17A^high^ and/or PDL-1^high^ cells.

**Table 1:** PDL1 and IL-17A expression in tumor tissues of 122 breast cancer patients

	ER^+^		ER^−^	
	high	low	high	low
PDL1	12 (18)*	52 (82)	48 (82)	10 (18)
IL-17A	13 (20)	51 (80)	46 (79)	12 (21)

Tumours with > 90 IL-17-producing or PDL1-producing cells (i.e. the mean of the cohort) were considered to be high; those with ≤ 90 infiltrating IL-17-producing cells or PDL1-producing cells were considered to be low.

*Numbers inside parentheses are percentages of patients.

The strong IL-17A and PDL1 expression in ER-negative tumor tissues prompted us to further investigate whether there was a positive correlation between IL-17A and PDL1 expression. qRT-PCR data showed that PDL1 mRNA was significantly increased in IL-17A^high^ tumor tissues (*p* < 0.05, Figure 1E). Meanwhile, IL-17A mRNA levels were significantly increased in PDL1^high^ tumor tissues (*p* < 0.05, Figure 1F). To confirm the correlation between IL-17A and PDL1 expression, we measured IL-17A in serum from PDL1^high^-expressing and PDL1^low^-expressing ER-negative cancer patients; IL-17A was increased in with PDL1^high^- as compared to PDL1^low^-expressing patients (*p* < 0.05, Figure 1G). These data suggested a positive correlation between IL-17A and PDL1 in ER-negative tumors.

### IL-17A promotes PDL1 expression through ERK phosphorylation in ER-negative cell lines, monocytes, and DCs

**Figure 2:**
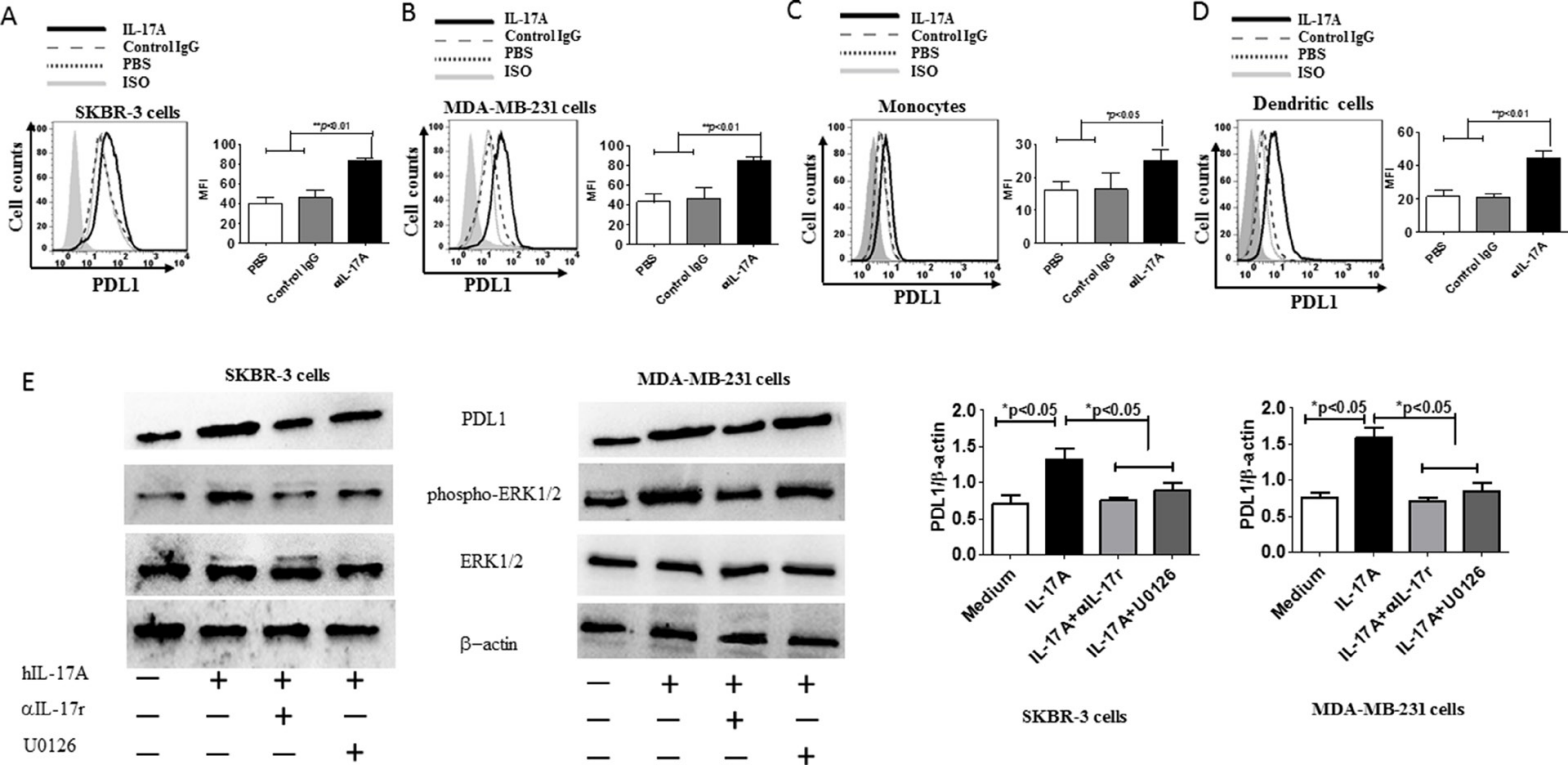
IL-17A promotes PDL1 expression on ER-negative cells, human monocytes, and DCs by ERK phosphorylation SKBR-3 cells (**A**) MDA-MB-231 cells (**B**) monocytes (**C**) and dendritic cells (**D**) were stimulated with protein IL-17A for 48 h, after which they were harvested and counted. The cells were washed and stained with anti-human PDL1 Ab and analyzed by flow cytometry. (**E**) Western blot analysis of PDL1, phospho(pT202/pY204)+Erk2(pT185/pY187) ERK1/2, and total ERK1/2 in SKBR-3 and MDA-MB-231 cell lines untreated (medium) or treated with 20 ng/ml of recombinant IL-17A, 20 ng/ml of recombinant IL-17A + 20 ng/ml anti-IL-17R, or 20 ng/ml of recombinant IL-17A + 20 mM U0126 for 3 h. Data are representative of three experiments. Error bars represent SEM.

To address whether PDL1^high^ was related with IL-17A, two ER-negative breast cancer cell lines, SKBR-3 and MDA-MB-231, were stimulated with IL-17A. As shown in Figure 2A and 2B, PDL1 expression was elevated by IL-17A stimulation in both cell lines. In addition to tumor cells, PDL1 is expressed on monocytes and DCs [16, 18]. Thus, we prepared monocytes and DCs from the patients and stimulated them with IL-17A. FACS analysis based on PDL1 staining showed that IL-17A promoted PDL1 expression in both cell types (Figure 2C and 2D).

Because IL-17A can increase breast cancer cell proliferation through the ERK1/2 pathway [6], to address the mechanism of PDL1^high^ expression by IL-17A regulation, we next tested whether it depended on the ERK1/2 pathway. To this end, SKBR-3 and MDA-MB-231 were stimulated with recombinant human IL-17A and subsequently incubated with anti-IL-17RA Ab or the MEK inhibitor U0126, which blocks ERK1/2 phosphorylation by inhibiting MAPKK activity. The results showed that both anti-IL-17RA and U0126 inhibited IL-17A-induced PDL1^high^ expression (Figure 2E).

### IL-17A promotes PDL1 expression in mouse cell lines, PBMCs, and macrophages

**Figure 3:**
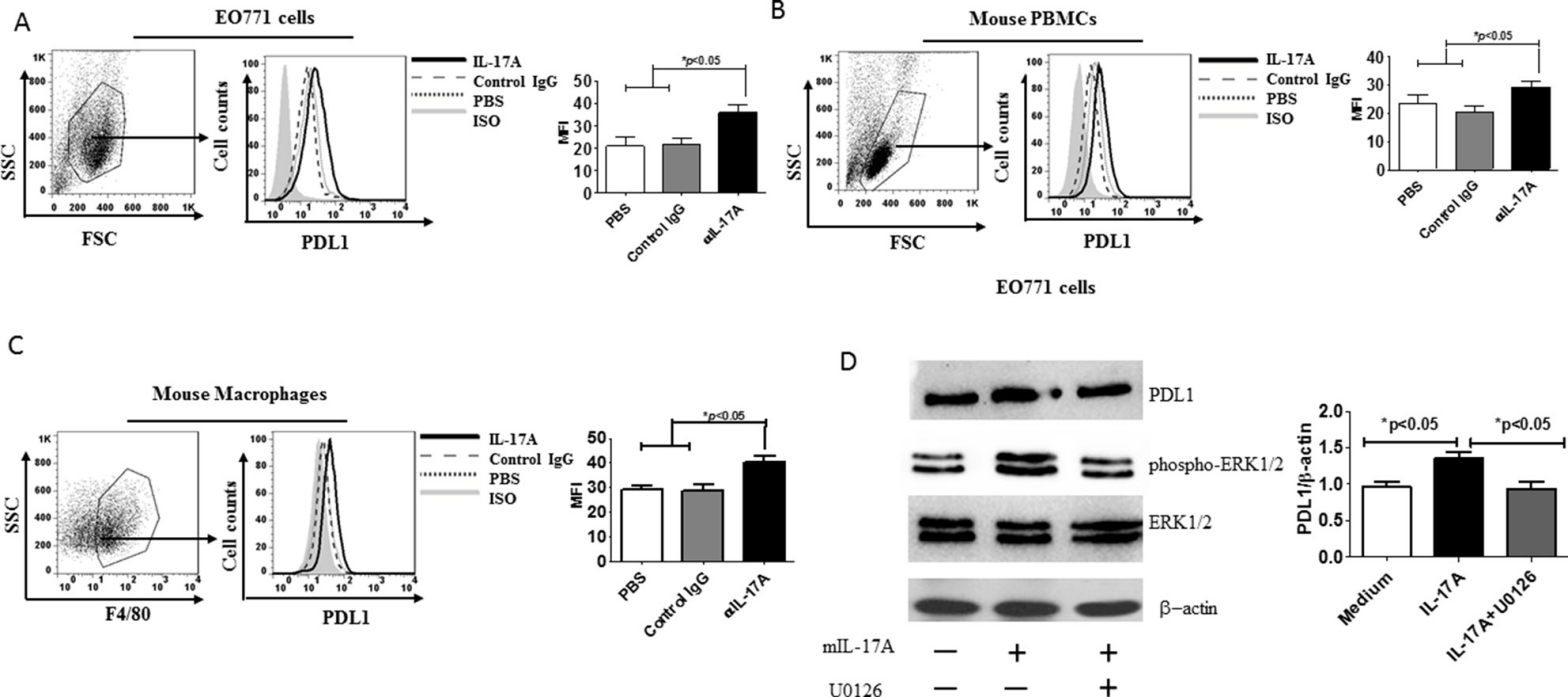
IL-17A promotes PDL1 expression in mouse cell lines, macrophages, and PBMCs EO771 cells (**A**) mouse PBMCs (**B**) and mouse macrophages (**C**) were stimulated with protein mouse IL-17A for 48 h, after which they were harvested and counted. The cells were washed and stained with anti-mouse PDL1 Ab and analyzed by flow cytometry. (**D**) Western blot analysis of PDL1, phospho(pT202/pY204)+Erk2(pT185/pY187) ERK1/2, and total ERK1/2 in EO771 cells treated or not (medium) with 20 ng/ml of recombinant mIL-17A or 20 ng/ml of recombinant mIL-17A + 20 mM U0126 for 3 h. Data are representative of three experiments. Error bars represent SEM.

Based on the findings in human cells, we next assessed whether mouse IL-17A also promotes PDL1 expression in EO771 mouse ER-negative breast cancer cells [19], macrophages, and PBMCs. IL-17A stimulation promoted PDL1 expression in the EO771 cells (Figure 3A) as well as the macrophages and PBMCs (Figure 3B and 3C). To address whether PDL expression also depends on ERK1/2 in the mouse cell line, EO771 cells were stimulated with recombinant mouse IL-17A and subsequently incubated with the MEK inhibitor U0126. The results showed that U0126 inhibited IL-17A-induced PDL expression (Figure 3D), suggesting that mouse IL-17A has a similar function as human IL-17A.

### Targeting of IL-17A lowers PDL1 expression in ER-negative mouse breast tumor tissues

**Figure 4:**
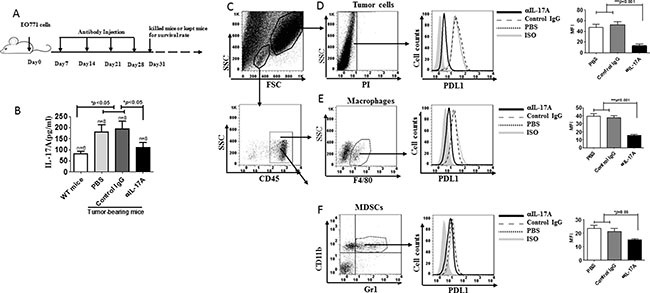
Decreased PDL1 expression in ER negative tumor-bearing mice following treatment with anti-IL-17A antibody (**A**) C57BL/6 mice were challenged with 1 × 10^6^ EO77 cells s.c. and treated with PBS or control IgG or anti-IL-17A Ab on different days. (**B**) Sera were harvested at 31 days post-tumor challenge for analyzing levels of the cytokines IL-17A. (**C**) The gating strategy of tumor cells and TILs from tumor-bearing mice. Tumors from tumor-bearing mice injected with PBS (*n* = 8), control IgG (*n* = 8), or anti-IL-17A mAb (*n* = 8) were harvested at 31 days post challenge and single cells were prepared. The viable cells were selected, and the tumor cells (**D**), macrophages (**E**), and MDSCs (**F**) were gated. PDL1 expression was analyzed by flow cytometry. Data are representative of three experiments. Error bars represent SEM.

EO771-bearing mice are a suitable model for immunotherapy studies [19, 20]. We established a murine tumor model by s.c. injection of EO771 cells and treated the mice with anti-IL-17A Ab to study the roles of IL-17A in tumor-bearing mice *in vivo* (Figure 4A). IL-17A levels were significantly increased in tumor-bearing mice treated with PBS or control IgG (*p* < 0.05, Figure 4B) as compared to wild-type mice. However, IL-17A was significantly lower in the anti-IL-17A-treated group than in the control groups (*p* < 0.05, Figure 4B). PDL1^high^ cells in tumor tissues play critical roles in breast cancer; they mediate immunosuppression and promote tumor development and progression [17, 21–23]. To address whether targeting of IL-17A could affect PDL1 expression in tumor tissues, we prepared single tumor cells from tumor-bearing mice treated with anti-IL-17A Ab and gated the tumor cells and live TILs to check PDL1 expression on tumor cells, macrophages, and MDSCs (Figure 4C). The data revealed that anti-IL-17A treatment decreased PDL1 expression on these cells (*p* < 0.05, Figure 4D–4F).

### Targeting of IL-17A induces an antitumor effect by transforming the tumor microenvironment

**Figure 5:**
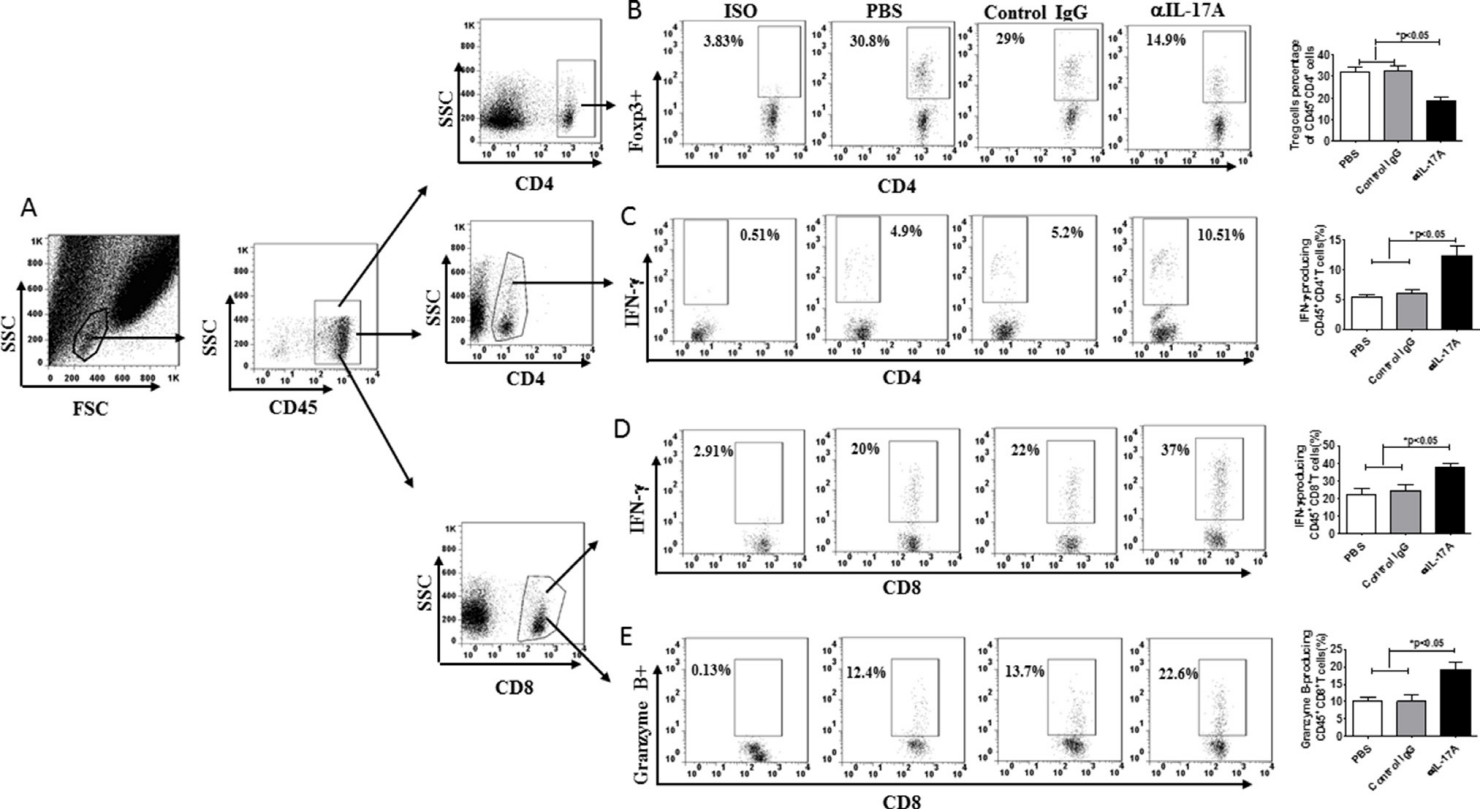
Anti-IL-17A antibody influenced the immunogenic tumor microenvironment (**A**) The gating strategy of TILs (Tumor infiltrating lymphocytes) from tumor-bearing mice. (**B**–**E**) C57BL/6 mice were challenged with 1 × 10^6^ EO771 cells s.c. and injected i.p. with anti-IL-17A antibody (200 mg/mouse, *n* = 8), control IgG (200 mg/mouse, *n* = 8) or PBS (*n* = 8) 7 days later, when palpable tumors formed (3–5 mm in diameter). Additional three times antibody treatments were administered to tumor-bearing mice every 7 days after EO771 inoculation. Tumors from the tumor-bearing mice were harvested at 31 days post-tumor challenge, and then the single cells were prepared. The viable cells were selected, and the TILs were gated. (B) Percentages of Treg cells within the CD45^+^CD4^+^ population in TILs. (C) Percentages of IFN-γ^+^ T cells within CD4^+^CD45^+^ TILs. (D) Percentages of IFN-γ^+^ T cells within CD8^+^CD45^+^ TILs. (E) Percentages of granzyme B+ T cells within CD8^+^CD45^+^ TILs. Data are representative of three experiments. Error bars represent SEM.

The strong effect of the anti-IL-17A Ab on PDL1 expression in murine tumor cells, macrophages, and MDSCs prompted us to further investigate whether targeting of IL-17A could transform the tumor microenvironment. To this end, we characterized TILs in anti-IL-17A Ab-treated tumors by flow cytometry to determine the potential roles of anti-IL-17A Ab-mediated tumor suppression in ER-negative breast cancer models (Figure 5A). The percentage of CD45^+^CD4^+^Foxp3^+^ cells was decreased in Ab-treated as compared to control-treated tumors (*p* < 0.05; Figure 5B). Most tumors can induce adaptive immune responses, and the presence of a higher number of IFN-γ- and granzyme B^+^-producing lymphocytes in tumor tissues correlates with better prognosis [24, 25]. We found that IFN-γ-producing CD8^+^ T cells, granzyme B-producing CD8^+^ T cells, as well as IFN-γ-producing CD4^+^ T cells were significantly increased in mice injected with anti-IL-17A as compared to control-treated mice (*p* < 0.05, Figure 5C–5E).

### Combined use of IL-17- and PDL1-targeting antibodies induces significant antitumor effects

**Figure 6:**
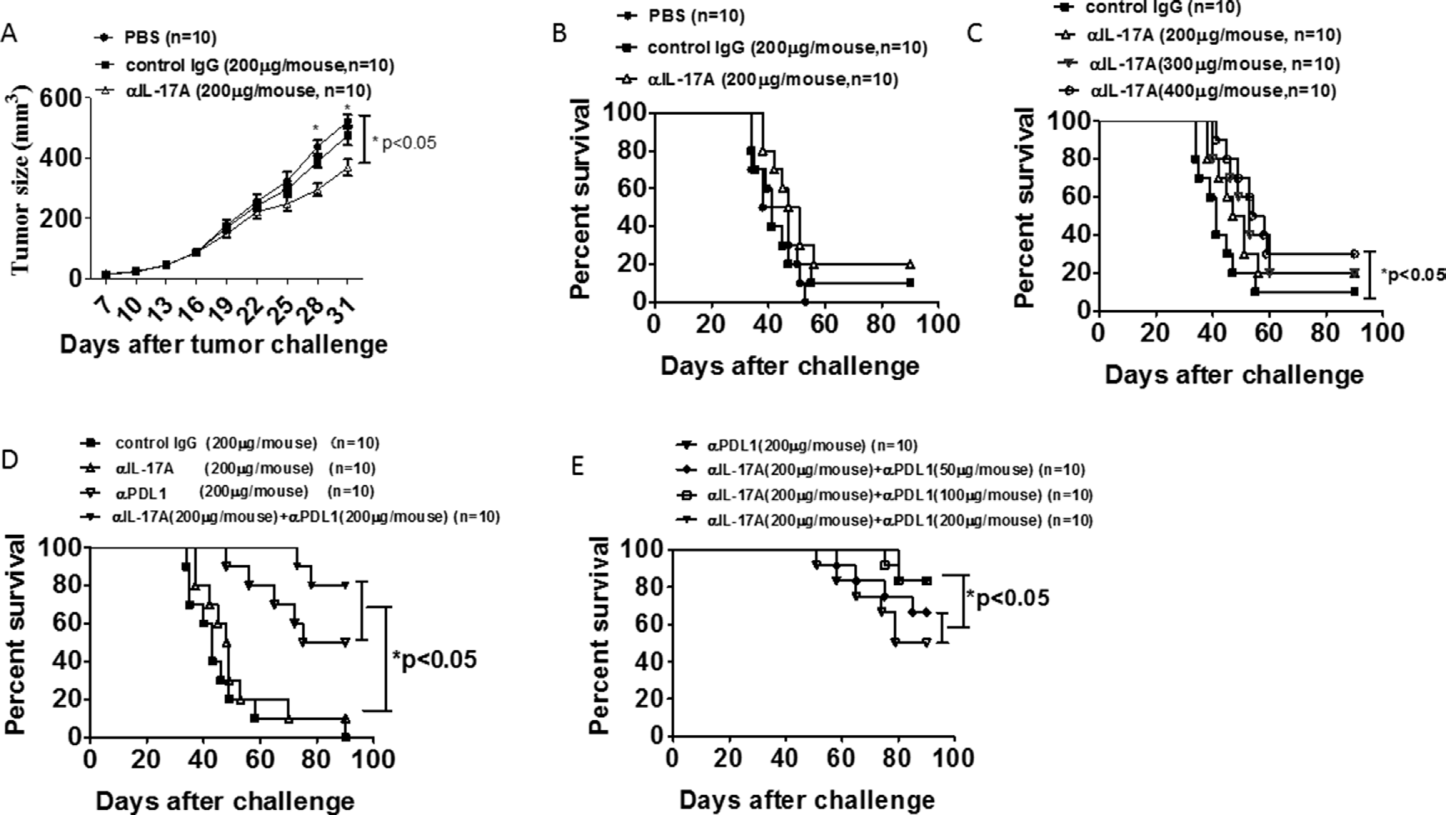
IL-17 and PDL1 antibody combined therapy reduced tumor burden and enhanced survival rate in ER negative tumor-bearing mice C57BL/6 mice were injected s.c. with 1 × 10^6^ CT-26 tumor cells. After 7 days, the tumor diameter reached 3–4 mm. The mice were injected four times at 1-week intervals with PBS, control IgG, anti-IL-17A antibody, and/or anti-PDL1 antibody at the indicated doses. Tumor growth (**A**) and survival rate (**B**–**E**) were recorded. Data are representative of three experiments. Error bars represent SEM.

Because targeting of IL-17A lowered PDL1 expression in tumor tissues and promoted the T cell response, especially of CD8^+^ T cells, in tumor-bearing mice, we next sought to determine whether antitumor immunity could be established by anti-IL-17A injection. Mice were first challenged with 1 × 10^6^ syngeneic EO771 tumor cells. At 7 days after inoculation, tumor-bearing mice were treated with anti-IL-17A Ab, control IgG, or PBS four times at 1-week intervals. As shown in Figure 6A, the tumor-bearing mice treated with anti-IL-17A had a significantly lower tumor burden than did mice treated with PBS or control IgG (*p* < 0.05). However, anti-IL-17A Ab treatment did not prolong the survival time of tumor-bearing mice (Figure 6B). Next, we increased the dose of IL-17A Ab to check whether targeting of IL-17A increased the survival time of tumor-bearing mice. We found that 400 mg IL-17A Ab/mouse prolonged the survival time of tumor-bearing mice (Figure 6C); however, high-dose antibody injections can cause serious side effects [26]. Because IL-17A Ab could decrease PDL1 expression in tumor tissues, we decided to treat the tumor-bearing mice with combined IL-17A and PDL1 Abs. The combined treatment significantly increased the survival rate as compared to the control and single-Ab treatments (Figure 6D). To confirm that IL-17A Ab injection could reduce the required dose of PDL1 Ab in tumor-bearing mice, IL-17A/PDL1 Abs were used at different dose ratios. As shown in Figure 6E, 50 mg/mouse PDL1 Ab combined with IL-17A Ab caused an approximately 70% increase in survival rate relative to that observed in the control mice. These data suggested that targeting of IL-17A is necessary for effective elicitation of the antitumor immune response by PDL1 Ab.

## DISCUSSION

In this study, we showed that IL-17A and PDL1 expression is increased and positively correlated in ER-negative breast cancer patients. Additionally, we established that IL-17A promotes PDL1 expression in human tumor cells, monocytes, and DCs, as well as in mouse tumor cells and macrophages. We found that anti-IL-17A Ab injection inhibits PDL1 expression in tumor cells, macrophages, and MDSCs, decreased the number of Treg cells in TILs, and increased the number of IFN-γ-producing CD4+ and CD8+ T cells in TILs in ER-negative tumor-bearing mice. Finally, we demonstrated the anti-tumor effect of combined IL-17/PDL1 Ab therapy.

In tumor microenvironments, inflammatory cytokines such as IFN-γ stimulate tumor cells to express PDL1 and inhibit the activity of tumor-specific T cells [12]. Our current study showed that IL-17A also promotes PDL1 expression. It is possible that ER-negative breast tumor cells increase PDL1 expression through IL-17A and IFN-γ to escape immune surveillance. It has been reported that recombinant IL-17A recruits the MAPK pathway by upregulating phosphorylated ERK1/2 in human breast cancer cell lines [6]. Our results corroborated that the MEK-ERK1/2 signal pathway participates in PDL1 expression regulated by IL-17A. However, blockage of ERK1/2 phosphorylation did not completely ablate the expression of PDL1; thus, we speculate that other pathways such as PI3K–Akt may participate in PDL expression regulated by IL-17A [14, 15].

In addition to tumor cells, PDL1 is commonly expressed on myeloid cells in the tumor microenvironment; blockage of PDL1 expression on DCs, macrophages, or MDSCs could inhibit tumor cell growth [27–30]. It is feasible to inhibit tumor cell growth by decreasing PDL1 expression with IL-17A Ab in the tumor microenvironment. Interestingly, while treatment with anti-IL-17 Ab lowered PDL1 expression in tumor cells, macrophages, and MDSCs, the expression level remained high; therefore, we speculated that IFN-γ plays a very important role in PDL1 upregulation in ER-negative breast cancer [12, 13]. Several studies have highlighted the importance of TILs in the regulation of cancer development and progression, including breast cancer [31, 32]. Our data revealed that the percentage of Treg cells in CD45^+^ cells of TILs reached almost 30% in control groups. However, this percentage was greatly decreased by anti-IL-17A mAb treatment. In addition, IL-17A mAb increased the number of IFN-γ− and granzyme B-producing CD8^+^ T cells. This is very important to transform the tumor microenvironment and enhance antitumor effects in favor of tumor eradication.

We demonstrated that targeting of IL-17A (200 mg/mouse) suppressed tumor growth but could not prolong the survival time of tumor-bearing mice. We speculate that this could be attributed to the high expression of PDL1 in tumor tissues. In breast cancer, blockade of PD1 or its ligand PDL1 by specific monoclonal antibodies has been shown to reverse this effect and to potentiate cancer therapeutic immunity [23, 33]. However, single blockade of immune checkpoint molecules (such as PD1) cannot effectively eliminate cancer cells [34, 35]. A combination with cancer immunotherapy may enhance the anti-tumor effect [34]. Combined IL-17A/PDL1 Ab injection significantly increased the survival rate in the current study, and IL-17A Ab injection could reduce the dose of PDL1 Ab in tumor-bearing mice. We speculate that combined IL-17A and PDL1 Abs may enhance the anti-tumor immune response against PDL1 Ab by decreasing PDL1 expression. This study also suggest that targeting of IL-17A is necessary for the antitumor immune responses elicited by PDL1 Ab. Our findings strongly suggest that IL-17A serves as an inflammatory cytokine to promote PDL1 expression, and targeting of IL-17A and PDL1 enhances tumor-specific immune responses and breaks tolerance to tumor antigens. These findings provide further evidence of the tumor suppressor function of IL-17A *in vivo*.

One limitation of our study lies in that we only investigated the role of IL-17A in ER-negative breast cancer. Out of 122 patients with breast cancer, 64 (52%) had ER-positive tumors; about 20% of ER-positive tumors contained IL-17A^high^ and/or PDL-1^high^ cells, indicating that high expression of IL-17A and PDL1 may not be related to ER status. In addition, it has been reported that inhibition of IL-17A could enhance the anti-tumor immune response in melanoma and lung cancer [10, 11]; thus, it is possible that targeting of IL-17A suppresses tumor growth in ER-positive breast cancer. Further studies are required to evaluate the combinatorial effect of IL-17A with immune components such as CTLA4 and other therapeutic agents, including chemotherapy and antibody-based therapies, in the context of animal models with intact immune systems. In conclusion, our study established a pro-tumor role of IL-17A that has important implications for ER-negative breast cancer and laid the foundation for further exploration of the potential of IL-17A as a target in cancer immunotherapy.

## MATERIALS AND METHODS

### Patients and sample collection

Breast cancer specimens and blood were collected from 122 female patients (Table 2) that underwent surgery in The First Affiliated Hospital of Xi’an Jiaotong University (China). The age of the patients ranged from 39 to 68 years. Individuals were deemed eligible for the study if they were primary breast cancer patients that had not received any prior treatment. In this study, we concentrated on breast cancer patients diagnosed with infiltrating ductal carcinoma, which is the most common subtype of breast cancer. Other tumor subtypes, including infiltrating lobular and medullary carcinomas, were excluded because they are very rare in the Chinese population. Patients presenting with additional conditions, such as other malignancies, or advanced organ failure, were also excluded. The patients examined were in clinical stages I, II, or III according to TNM classification. Clinical diagnosis was routinely confirmed by histopathological examination of the tumor tissue samples. ER, progesterone receptor, and HER2 status was determined by immunohistochemistry. This study conformed to the ethical standards of the World Medical Association Declaration of Helsinki and was approved by the Ethics Committee of The First Affiliated Hospital of Xi’an Jiaotong University School of Medicine (approval number: xjtu20150824). Signed informed consent was obtained from all patients.

**Table 2:** Demographic data of recruited patients with breast cancer

	Age(Years)	Numbers
**Healthy Subjects**	28–57	30
**Ductal Carcinoma**	35–65	122
HER2 Positive	35–63	37
PR Positive	37–65	62
ER Positive	37–65	64
I stage	35–47	35
II stage	30–59	50
III stage	34–65	37

### Mice and cell lines

Eight-week-old female C57BL/6 mice were purchased from the Animal Experimentation Center of Xi’an Jiaotong University, China. All mice used in the experiments were housed under specific pathogen-free conditions in the animal facility at Xi’an Jiaotong University and treated in accordance with the guidelines of the Institutional Animal Care and Use Committee (approval number: xjtu20150913). The human ER-negative breast cancer cell lines MDA-MB-231 and SKBR-3 [36], and mouse ER-negative breast cancer cells EO771 [19], obtained from the American Type Culture Collection, were maintained in DMEM supplemented with 10% heat-inactivated FCS, 2 mM l-glutamine, 100 U/ml penicillin, and 100 mg/ml streptomycin.

### Cytokine-specific ELISA

The serum IL-17A concentrations of breast cancer patients, healthy donors, and tumor-bearing mice were measured via ELISA. In briefly, serum samples were harvested and incubated in culture plates coated with rabbit anti-human IL-17A or anti-mouse IL-17A polyclonal antibody (1:500; Abcam) at 4°C overnight, washed with PBS containing 0.05% Tween-20, and blocked with goat serum (1:10) at 37°C for 2 h. Then, 100 ml of serum was added to each well and incubated at room temperature for 2 h. After washing with PBS five times, mouse anti-human IL-17A mAb or goat anti-mouse IL-17A mAb (10 mg/ml in PBS; Abcam) was added to each well and incubated at room temperature for 1 h. HRP-conjugated goat anti-mouse IgG (1:4000) or rabbit anti-goat IgG was added to each well following washing with PBS and incubated at room temperature for 1 h. The wells were washed five times and substrate solution (100 ml of 0.1 M sodium phosphate citrate buffer containing 0.5 mg/ml *o*-phenylenediamine and 0.003% H_2_O_2_, pH 5.0) was added. The reaction was stopped with 50 ml of 2 N H_2_SO_4_ and the absorbance at 450 nm was measured.

### Immunohistochemistry (IHC)

IL-17A and PDL1 expression in cancer tissues were determined immunohistochemically by analyzing paraffin-embedded specimens fixed in 4% buffered formalin. Slides containing tumor sections were incubated for 30 min in citrate buffer (pH 6.0) at 96°C, followed by a 1-h incubation at room temperature with rabbit anti-human IL-17A or PDL1 polyclonal Ab (1:40; Abcam). The sections were further incubated with HRP-labeled anti-rabbit IgG (1:500; Abcam) and developed using 3,3′-diaminobenzidine tetrahydrochloride. For quantification, the IL-17- and PDL1-stained cells were manually counted in five high-power fields per tumor tissue at 400× magnification. Strong staining for IL-17 or PDL1 was considered to indicate positivity, and areas with the highest number of stained cells were selected for scoring. The average number of IL-17- or PDL1-stained cells in each tumor tissue was considered to be representative of IL-17- or PDL1-producing cells in the tumor. Tumors with > 90 and ≤ 90 IL-17/PDL1-producing cells were considered high- and low-positive, respectively.

### Quantitative reverse transcription polymerase chain reaction (qRT-PCR)

Total RNA was extracted from the tumor tissue samples by using TRIzol (Invitrogen, Shanghai, China). A RevertAid^TM^ First-Strand cDNA Synthesis Kit (Fermentas, Shenzhen, China) was used to synthesize cDNA from 250 ng of RNA. cDNA was amplified in a 25-μL reaction mixture containing 12.5 μL of SYBR Green Supermix (Bio-Rad Beijing, China), 100 ng of cDNA template, and qPCRs were performed with a SYBR Green qPCR kit (Invitrogen) on an iCycler system (Bio-Rad) with the primer sets listed in Table 3.

**Table 3:** Primer sequences

	Forward	Reverse
IL-17A	CGGACTGTGATGGTCAACCT	GAGCTCTTAGGCCACATGGT
PDL1	CTGGCATTTGCTGAACGCAT	GGGAGAGCTGGTCCTTCAAC
GAPDH	ACCACAGTCCATGCCATCAC	TCCACCACCCTGTTGCTGTA

### Western blotting

SKBR-3, MDA-MB0231, and EO771 cells were seeded in 6-well plates at 3 × 10^6^ cells/well and cultured overnight in FCS-free medium. The cells were stimulated with human or mouse IL-17A at 20 ng/ml and/or U0126 (Sigma, Shanghai, China) at 10 mM, and anti-human IL-17A-receptor Ab (Abcam) at 20 ng/ml for 3 h. Then, the medium was removed and the cells were lysed in 1% Triton X-100 lysis buffer, incubated on ice for 1 h, and centrifuged at 4°C for 15 min at 10,000 × *g*. Supernatants were collected and the protein concentration was determined using the Bradford assay (Bio-Rad, Beijing, China). Immunoblotting was carried out with rabbit anti-PDL1, anti-Erk1(pT202/pY204)+Erk2(pT185/pY187), anti-ERK1+ERK2, or anti-β-actin (Abcam) Ab. The blots were visualized using the ECL Prime WB detection reagents (GE Healthcare Biosciences, Pittsburgh, PA).

### Preparation of monocyte-derived dendritic cells (DCs)

Peripheral blood mononuclear cells (PBMCs) were isolated from blood samples of the patients by fractionation over Ficoll (Invitrogen) gradients. To obtain monocytes, cells were enriched for monocytes by positive selection with mAb to CD14 (CD14^+^ monocytes Isolation Kit; Miltenyi Biotec, Shanghai, China). The monocytes were cultured in 6-well plates at 3 × 10^6^ cells/well in RPMI1640 medium (Gibco, Beijing, China) containing 10% heat-inactivated FBS and supplemented on days 0, 2, and 4 with 1000 U/ml IL-4 (PeproTech, Suzhou, China) and 800 U/ml GM-CSF (PeproTech). DC maturation was accomplished by replating the cells for 2 days in 6-well plates at 2 × 10^6^ cells/well in IL-4 (1000 U/ml) and GM-CSF (800 U/ml) supplemented with IL-1β (10 ng/ml; PeproTech), TNF-α(10 ng/ml; BD Pharmingen), IL-6 (1000 U/ml; BD Pharmingen), and PGE_2_ (1 mg/ml; Sigma-Aldrich). Mature DCs were consistently 90–95% CD11c^+^DR^+^ with < 2% CD14^+^ cells.

### Fluorescence-assisted cell sorting (FACS) analyses

Assay-ready SKBR-3 and MDA-MB-231 cells (3 × 10^6^ cells/well) were treated with 20 ng/ml IL-17A (R&D, Beijing, China) for 48 h, after which they were harvested and counted. Five microliters of FITC anti-PD-L1 (BioLegend, Beijing, China) was added for 30 min at 4°C. The cells were washed and stained with PI (Invitrogen) and analyzed by flow cytometry.

Human PBMCs were isolated from ER(-) patient blood samples by adherence to plastic for 1 h and extensively washed to remove nonadherent cells. The monocytes were subsequently seeded in 6-well plates at 3 × 10^6^ cells/well and cultured with 20 ng/ml IL-17A. The cells were harvested and stained with anti-PDL1 Ab for FACS analysis. Monocyte-derived DCs were stained following the same basic procedure.

Murine macrophages were harvested from peritoneal exudates 48 h after i.p. injection of liquid 5% thioglycolate medium (BD Biosciences, Beijing, China). Mouse PBMCs were harvested from C57BL/6 mice. The mouse ER(-) breast cancer cell line EO771, macrophages and PBMCs were stimulated with 20 ng IL-17A/ml for 48 h and stained with anti-mouse F4/80 and/or anti-mouse PDL1. Mean fluorescence intensity (MFI) was determined by gating on live cells and subtracting background (isotype) MFI.

### Tumor challenge

To generate a murine model of ER-negative breast cancer, C57BL/6 mice were challenged with 1 × 10^6^ EO771 cells subcutaneously (s.c.) and injected intraperitoneally (i.p.) with anti-IL-17A Ab and/or anti-PDL1 Ab at the indicated doses 7 days later, when palpable tumors formed (3–5 mm in diameter). Three additional antibody treatments were administered to tumor-bearing mice every 7 days after EO771 inoculation. Tumor volumes were measured every 2 to 3 days with an electronic caliper and calculated with the formula (L1^2^ × L2)/2, where L1 is the shortest diameter and L2 is the longest diameter. Mice were sacrificed when tumors grew to 15 mm in diameter; in some experiments, the mice were sacrificed on day 31 after EO771 inoculation, and surface and intracellular cytokine-staining patterns of macrophages, MDSCs, and T cells were analyzed by flow cytometry.

### Surface and intracellular molecular staining

For PDL1 staining of tumor cells, tumor-associated macrophages, and MDSCs, tumor tissue samples from tumor-bearing mice injected with anti-IL-17A Ab, control IgG, or PBS were weighed and minced and subjected to digestion with a triple enzyme mixture containing collagenase type IV, hyaluronidase, and deoxyribonuclease for 45 min at 37°C on a rotating platform. Single cells were surface-stained with PI, anti-CD45, anti-CD11b, anti-Gr1 or anti-F4/80, anti-PDL1 (BioLegend); the live cells were gated and analyzed by flow cytometry.

For intracellular staining of tumor tissue, single-cell suspensions of tumor tissue were stimulated with 50 ng/mL PMA and 100 ng/mL ionomycin in the presence of GolgiPlug (BD, Beijing, China). After 4 h, the cells were surface-stained with anti-CD4 or anti-CD8 mAbs, fixed, permeabilized, stained with anti-IFN-γ, anti-granzyme B, and anti-Foxp3 mAbs (BioLegend), and washed. The tumor-infiltrating lymphocytes (TILs) were gated and analyzed by flow cytometry.

### Statistical analyses

The data were evaluated using ANOVA followed by Tukey's multiple comparison test for more than two study groups. Kaplan–Meier survival analysis was used to determine the significance of the observed differences in results for *in vivo* tumor therapy. Values of *p* < 0.05 (*), *p* < 0.01 (**), and *p* < 0.001 (***) were considered statistically significant.

## Abbreviations

ER: estrogen receptor
HER2: human epidermal growth factor receptor 2
IFN-γ: interferon-γ
IL-17: interleukin-17
MDSCs: myeloid-derived suppressor cells
PBMCs: peripheral blood mononuclear cells
PD1: programmed cell death 1
PDL1: programmed death ligand 1
PR: progesterone receptor
TILs: tumor-infiltrating lymphocytes
